# WTAP-mediated m^6^A modification of lncRNA Snhg1 improves myocardial ischemia-reperfusion injury via miR-361-5p/OPA1-dependent mitochondrial fusion

**DOI:** 10.1186/s12967-024-05330-4

**Published:** 2024-05-25

**Authors:** Linlin Liu, Jiahong Wu, Cheng Lu, Yan Ma, Jiayi Wang, Jie Xu, Xiaoli Yang, Xuan Zhang, Hua Wang, Jieyu Xu, Jiehan Zhang

**Affiliations:** Department of Cardiology, Seventh People’s Hospital of Shanghai University of Traditional Chinese Medicine, No. 358, Datong Road, Pudong New Area, Shanghai, 200137 China

**Keywords:** LncRNA Snhg1, miR-361-5p, OPA1, WTAP, MIRI, Apoptosis, Mitochondrial polarization

## Abstract

**Background:**

Myocardial ischemia-reperfusion injury (MIRI) is caused by reperfusion after ischemic heart disease. LncRNA Snhg1 regulates the progression of various diseases. N6-methyladenosine (m^6^A) is the frequent RNA modification and plays a critical role in MIRI. However, it is unclear whether lncRNA Snhg1 regulates MIRI progression and whether the lncRNA Snhg1 was modified by m^6^A methylation.

**Methods:**

Mouse cardiomyocytes HL-1 cells were utilized to construct the hypoxia/reoxygenation (H/R) injury model. HL-1 cell viability was evaluated utilizing CCK-8 method. Cell apoptosis, mitochondrial reactive oxygen species (ROS), and mitochondrial membrane potential (MMP) were quantitated utilizing flow cytometry. RNA immunoprecipitation and dual-luciferase reporter assays were applied to measure the m^6^A methylation and the interactions between lncRNA Snhg1 and targeted miRNA or target miRNAs and its target gene. The I/R mouse model was constructed with adenovirus expressing lncRNA Snhg1. HE and TUNEL staining were used to evaluate myocardial tissue damage and apoptosis.

**Results:**

LncRNA Snhg1 was down-regulated after H/R injury, and overexpressed lncRNA Snhg1 suppressed H/R-stimulated cell apoptosis, mitochondrial ROS level and polarization. Besides, lncRNA Snhg1 could target miR-361-5p, and miR-361-5p targeted OPA1. Overexpressed lncRNA Snhg1 suppressed H/R-stimulated cell apoptosis, mitochondrial ROS level and polarization though the miR-361-5p/OPA1 axis. Furthermore, WTAP induced lncRNA Snhg1 m^6^A modification in H/R-stimulated HL-1 cells. Moreover, enforced lncRNA Snhg1 repressed I/R-stimulated myocardial tissue damage and apoptosis and regulated the miR-361-5p and OPA1 levels.

**Conclusion:**

WTAP-mediated m^6^A modification of lncRNA Snhg1 regulated MIRI progression through modulating myocardial apoptosis, mitochondrial ROS production, and mitochondrial polarization via miR-361-5p/OPA1 axis, providing the evidence for lncRNA as the prospective target for alleviating MIRI progression.

## Background

Ischemic heart disease (IHD) is a main cause of death around the world, with high mortality and morbidity [1, 2]. Myocardial infarction is the most popular primary symptom of IHD [3]. In clinical therapy, prompt reperfusion is crucial to decrease infarct size, save the ischemic myocardium, and prevent the onset of heart failure [1, 4]. Nevertheless, reperfusion may induce myocardial injury named myocardial ischemia/reperfusion injury (MIRI) [4–6]. Increasing evidence reveals that reperfusion injury accounts for 50% of the approximate myocardial infarct size [7, 8]. MIRI leads to significantly increased mortality in myocardial infarction [9]. Thus, MIRI is a primary risk factor threatening global health. It is significant to clarify the mechanism of the MIRI progression and explore the potential therapy strategy to limit myocardial injury.

Mitochondrial dysfunction is closely related to metabolic disorders, ischemic heart disease, and many other diseases [10]. During MIRI, mitochondria ATP production and mitochondrial membrane potential (MMP) are decreased, while reactive oxygen species (ROS) are excessively produced, which collectively lead to myocardial dysfunction, DNA damage, and apoptosis [11]. In the process of apoptosis, mitochondrial fusion is observed, which is regulated by several proteins including optic atrophy protein 1 (OPA1) and mitofusin 1 (MFN1) and 2 (MFN2) [12]. Therefore, it is important to identify potential factorsthat modulate mitochondrial dynamics to prevent MIRI.

Long non-coding RNAs (lncRNAs) belong to the non-coding RNAs (ncRNA) family [13]. The lncRNAs are over 200 nt in length without protein-coding capacity but usually modulate gene expressions at the post-transcriptional level via competing endogenous RNAs (ceRNAs) mechanism [14]. The ceRNA mechanism proposes that lncRNAs may serve as ceRNA sponges to absorb microRNAs (miRNAs), thereby mitigating the inhibitory action of miRNAs on target mRNAs [15]. As a member of the lncRNAs family, lncRNA SNHG1 has been extensively studied in a variety of diseases, including MIRI. For example, lncRNA SNHG1 in HL-1 cells was significantly reduced by H/R treatment and assuaged HL-1 cell pyroptosis by regulating the KLF4/TRPV1/AKT axis through sponging miR-137-3p [16]. lncRNA SNHG1 expression was downregulated in H/R-induced H9c2 cells and SNHG1/miR-16-5p/GATA4 regulated H9c2 cell apoptosis induced by H/R [17]. lncRNA SNHG1 expression in H/Rinduced AC16 cells was significantly decreased and SNHG1/miR-450b-5p/IGF1 axis inhibited the apoptosis and oxidative stress levels of H/Rinduced AC16 cells [18]. Moroever, knockdown of SNHG1 alleviated mitochondrial dysfunction in cerebral ischemia/reperfusion injury [19]. However, whether lncRNA SNHG1 may regulate cardiomyocyte apoptosis and mitochondrial dysfunction in MIRI progression by sponging miRNA is still poorly understood.

N6-methyladenosine (m^6^A) is the frequent RNA modification in eukaryotic cells at the posttranscriptional level [20, 21]. The potential regulatory efect of m6A modifcation may infuence the occurrence and development of a variety of cardiovascular diseases [22]. Studies have shown that Wilms tumor 1-associated protein (WTAP) is a RNA methyltransferase that promotes myocardial I/R injury progression and mediates lncRNA m6A modifcation through m^6^A reader [23, 24]. Prediction analysis by SRAMP database revealed multiple m6A modifcation sites in lncRNA SNHG1 sequence. However, whether lncRNA SNHG1 was modified by WTAP-mediated m^6^A methylation in MIRI is still poorly understood.

This study was therefore conducted to investigate the role of WTAP-mediated m^6^A methylation of lncRNA Snhg1 in myocardial injury triggered by mitochondrial dysfunction after MIRI. We showed for the first time that lncRNA Snhg1 sponged miR-361-5p to upregulate the expression of OPA1, which then activated the mitochondrial fusion to attenuate MIRI. Moreover, WTAP induced lncRNA Snhg1 m6A modification and inhibited lncRNA Snhg1 stability by m^6^A reader YTH N6-methyladenosine RNA-binding protein 2 (YTHDF2). The results of the present study may provide a theoretical basis for future clinical studies and highlight potential targets for the treatment of MIRI.

## Materials and methods

### Cell culture

Mouse cardiomyocytes HL-1 cells were maintained in Claycomb medium (Sigma Aldrich) with addition of 10% FBS (GIBCO, Grand Island, NY, USA) and 1% Penicillin–Streptomycin (Solarbio, Beijing, China) at 37 °C with 5% CO_2_.

### Hypoxia/Re-oxygenation (H/R) model

H/R model was constructed using HL-1 cells [25]. Briefly, cells were maintained in medium without serum at hypoxic condition (94% N_2_, 1% O_2_, and 5% CO_2_) for 6 h. Then, cells was replaced with fresh normal medium and maintained at normal condition (21% O_2_ and 5% CO_2_) for 12, 24, or 48 h to simulate reoxygenation injury.

### Cell transfection

pLVX-Puro-Snhg1 (oeSnhg1), pLVX-Puro-Wtap (oeWtap), pLKO.1-Snhg1 shRNA (shSnhg1), pLKO.1-Wtap shRNA (shWtap), pLKO.1-Opa1 shRNA (shOpa1) and pLKO.1-Ythdf2 shRNA (shYthdf2) vectors were constructed by GENERAL BIO (Chuzhou, China). The respective blank plasmids were designated as negative controls. Transfection was achieved though applying Lipofectamine 2000 (Invitrogen, Waltham, MA, USA) as per the supplier’s procedures. Forty-eight hours later, viral supernatants were obtained and infected HL-1 cells. The miR-361-5p mimic, inhibitor, and negative control (miNC) were provided by Genepharm (Suzhou, China).

### Cell viability assay

Forty-eight hours after treatment, cells were dispensed into 96-well plates with 3 × 10^3^ cells each well. Twenty-four hours later, cells were added to 10 µL of CCK-8 for 1 h incubation. The absorbance was recorded at 450 nm utilizing the microplate reader.

### Flow cytometry

HL-1 cells were dispensed into a six-well plate for 24 h cultivation. After treatment, cells were performed centrifugation (1,000×g, 5 min) and then treated with Annexin V-FITC (5 µL, 15 min) and propidium iodide (5 µL, 5 min). The apoptotic cells were measured using a flow cytometer (Becton-Dickinson FACS Calibur, San Joes, CA, USA). Mitochondrial ROS levels were assessed using a MitoSOX probe [26]. After treatment, 5 µM MitoSOX Red (Invitrogen) was introduced to HL-1 cells for 30 min incubation and cells were measured utilizing flow cytometry. Moreover, the mitochondrial membrane potential (MMP) ratio was designated as red (JC-1 aggregates)/green (JC-1 monomers) fluorescence intensity utilizing a JC-1 assay kit (C2006, Beyotime, Jiangsu, China) and analyzed utilizing a flow cytometry.

### Measurement of ATP

ATP level was evaluated using an ATP assay kit (Jiancheng, Nanjing, China) following the supplier’s instructions. HL-1 cells were plated into the 6-well plate for24 h. After that, HL-1 cells were collected utilizing lysis buffer. The detection solution was then introduced to the supernatant and ATP level was tested via normalizing to the total protein content.

### Quantitative real-time PCR (RT-qPCR)

RNAs samples of HL-1 cells were acquired utilizing Trizol (Invitrogen) and produced cDNA utilizing a RevertAid First Strand cDNA Synthesis Kit (Fermentas, Glen Burnie, MD, USA). RT-qPCR was conducted through applying Maxima SYBR Green/ROX qPCR Master Mix (2X) (Thermo Fisher Scientific, Waltham, MA, USA) as previously described [27]. The PCR primers were presented in Table 1. Results were quantitated with the 2^− ΔΔCt^ by normalizing to *GAPDH* or *U6*.

**Table 1:** Primer sequence information

Name	Forward	Reverse
*Snhg1*	5’-TGCTTGTAGTCAGGGTGCTG-3’	5’-AACACTGGCCTGGACAAACA-3’
*Opa1*	5’-CTGCAGGTCCCAAATTGGTT-3’	5’-CCCGCACTGAGTGGGTTTAT-3’
*Wtap*	5’-ATCAGGCAGAGGTCACAAGC-3’	5’- GGGTGGGCTTCAGCAGTAAT-3’
*Ythdf2*	5’-CAGGCATCAGTAGGGCAACA-3’	5’-AGTAGATCCAGAACCCGCCT-3’
*Gapdh*	5’-CTGCCCAGAACATCATCC-3’	5’-CTCAGATGCCTGCTTCAC-3’.
*miR-361-5p*	5’-GCGCGTTATCAGAATCTCCAG-3’	5’-AGTGCAGGGTCCGAGGTATT-3’
*U6*	5’-GCTTCGGCAGCAC-3’	5’-GGAACGCTTCACG-3’

### Protein preparation and western blot assay

Proteins samples were obtained with RIPA buffer plus protease inhibitor cocktail (Sigma, Louis, MO, USA). Equivalent quantities (20 µg) of proteins were run on 10% SDS-PAGE gels and transferred onto nitrocellulose membranes. After obstructed non-specific bindings, the membranes were probed to respective detecting antibodies target OPA1 (Abcam; ab157457; 1:1000), WTAP (Abcam; ab195380; 1:1000), YTHDF2 (Abcam; ab220163; 1:1000), or GAPDH (Proteintech; 10494-1-AP; 1:8000) at 4 °C for 12 h, followed by dyed with with secondary antibodies (ZSGB-BIO, Beijing, China; ZB-2301, ZB-2305; 1:10000) at 37 °C for 1 h. The blots were observed utilizing Immobilon Western Chemiluminescent HRP Substrate (Millipore, Bedford, MA, USA). The intensities of bands were quantitated using Image J (NIH, Bethesda, MD, USA).

### Analysis of m^6^A content

RNAs samples were acquired utilizing Trizol (Invitrogen). The purification of Poly(A)^+^ RNA was performed utilizing GenElute™ mRNA Miniprep Kit (Sigma, Louis, MO, USA). The m^6^A level was estimated utilizing m^6^A RNA Methylation Assay Kit (Abcam; ab185912) as previously described [28].

### RNA immunoprecipitation (RIP)

RIP experiment was conducted through applying the Magna RIP RNA-Binding Protein Immunoprecipitation kit (Millipore) as previously described [28]. HL-1 cell lysates were obtained utilizing RIP reagent and the RNA-protein complexes were probed to anti-AGO2 (Abcam; ab186733), anti-m6A (Abcam; ab208577), anti-YTHDF2 (Abcam; ab220163) or anti-IgG antibody (Abcam; ab172730) overnight at 4 °C. After washing step using RIP-wash buffer and RIP-lysis buffer, the adsorbed RNAs were obtained utilizing phenol: chloroform: isoamyl alcohol and analyzed by RT-qPCR.

### Dual-luciferase reporter assay

Putative target regions of miR-361-5p to lncRNA Snhg1 and Opa1 3’-UTR were predicted using Starbase and TargetScan. Predicted sequences of both wild type and mutant were synthesized, respectively, and then inserted into luciferase reporter vectors (PmirGLO), the vectors were then nominated as WT 3’-UTR and Mut 3’-UTR. The pGL3-lncRNA Snhg1 or pGL3-Opa1 3’-UTR and pRL-TK renilla (Promega) luciferase reporter vector were introduced into HL-1 cells accompanied by miR-361-5p mimic or inhibitor. The sequence of lncRNA Snhg1 containing the m^6^A motifs was generated by Generay Technologies (Shanghai, China) and cloned into the upstream of the pGL3-basic firefly luciferase vector. After that, the pGL3-lncRNA Snhg1 and pRL-TK renilla were co-transfected into the HL-1, which were stimulated by H/R and introduced into Wtap silencing or Wtap-expressing plasmid. The luciferase activities were recorded utilizing the dual luciferase reporter gene kit (Yuanpinghao, Beijing, China) 48 h after transfection.

### Determination of mRNA stability

HL-1 cells were incubated with 0.2 mM actinomycin D (Selleck, Shanghai, China). At the time point of 0, 3, and 6 h, total RNAs were isolated and produced cDNA utilizing the oligo(dT) primer. The mRNA expressions were quantified utilizing RT-qPCR.

### Animal experiment and drug administration

C57/BL6 mice aged 6–7 weeks (male, 20–25 g) were obtained from the SLAC Laboratory Animal Center of Shanghai (Shanghai, China). The mouse myocardial I/R model was constructed following previously described [29]. Recombinant adenovirus expressing lncRNA Snhg1 (2 × 10^10^ pfu/ml) or blank pShuttle-CMV vector was introduced into the left ventricular anterior wall via injection 24 h prior to I/R (four injections of 30 µl each with an interval of 4′30″ between injections). At 24 h after I/R, the cardiac function was evaluated using the Vevo 2100 Imaging (Visual Sonics, Toronto, Canada) echocardiographic system equipped with a 30 MHz transducer and left ventricular ejection fraction and left ventricular fractional shortening were calculated [30]. Heart rate, left ventricle end-diastolic pressure and left ventricle end-systolic pressure were recorded using an AcqKnowledge version 3.8.1 system (BIOPAC Systems, Inc. Goleta, CA, USA) with a sampling rate of 2,000 Hz [31]. All animal procedures were performed in adherence with the Guide for the Care and Use of Laboratory Animals published by the US National Institutes of Health (NIH Publication No. 85 − 23, 1996, revised 2011), and approved by the Ethical Committee of the Seventh People’s Hospital of Shanghai University of Traditional Chinese Medicine.

### Histological analysis and TUNEL staining

The myocardium was excised to conduct hematoxylin and eosin (HE) and terminal-deoxynucleoitidyl transferase mediated nick end labeling (TUNEL) staining as previously described [32]. Heart tissues were fixed, embedded, and cut into 5-µm sections. The sections were placed on glass slides, deparaffinized, and stained sequentially with hematoxylin and eosin (RichardAllan Scientific Co., Kalamazoo, MI, USA). The stained tissue sections were analyzed using a light microscope (Axio Imager M1; Carl Zeiss AG). Paraffin-embedded heart sections were used for apoptosis determination using TUNEL staining. Briefly, the slides were first incubated in 50 µg/mL proteinase K solution at 37 °C for 30 min. Then, followed by three times washing in PBS, slides were incubated with the TUNEL detection buffer at 37 °C for 1 h. Finally, the images were obtained and analyzed using the Olympus Fluoview FV300 version.

### Measurement of CK-MB and cTnT levels

The serum levels of mouse creatine kinase myocardialband (CK-MB; X-Y Biotechnology; XY9M0929) and myocardial troponin-T (cTnT; X-Y Biotechnology; XY9M0582) were estimated using ELISA kits and analyzed to assess the degree of myocardial damage.

### Data analysis

All experiments and assays were conducted with three replicates, and quantitative data were described as the mean ± standard deviation. Data analysis was achieved utilizing GraphPad Prism 8.4.2. Unpaired *t*-test and one-way ANOVA followed by Dunnett’s multiple comparisons test were selected to confirm group differences. *P* < 0.05 was designated as statistically significant.

## Results

### Overexpressed LncRNA Snhg1 restrained H/R-stimulated cell apoptosis, mitochondrial ROS production, and mitochondrial polarization

**Fig. 1:**
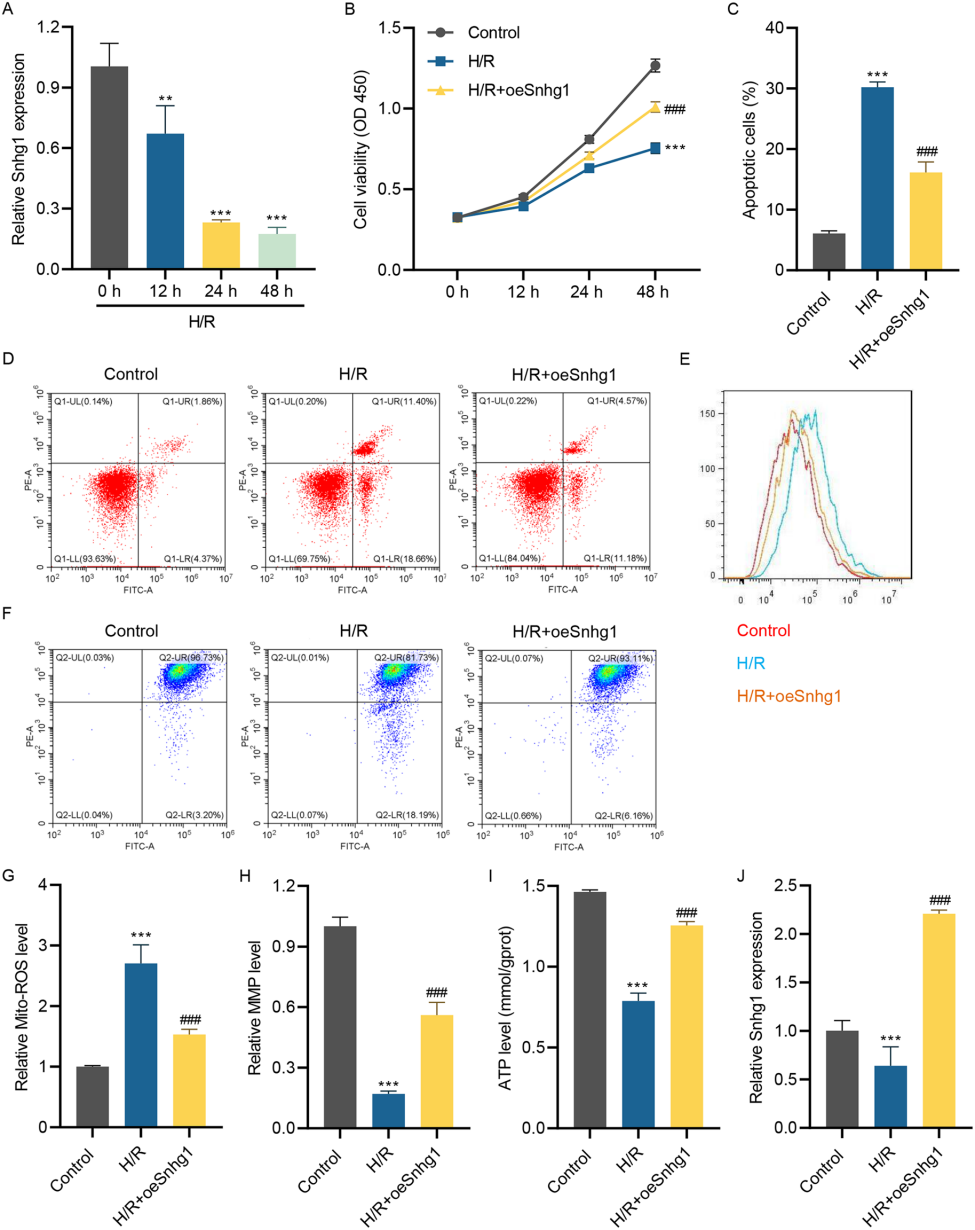
Overexpressed lncRNA Snhg1 restrains H/R-stimulated cell apoptosis, mitochondrial ROS production, and mitochondrial polarization. **(A)** The lncRNA Snhg1 level in HL-1 cells after 6 h of hypoxia and 0, 12, 24, or 48 h of re-oxygenation was measured using RT-qPCR. After HL-1 cells pre-transfected with lncRNA Snhg1 overexpressing lentivirus for 24 h and then received H/R challenge for 24 h, cell viability **(B)**, cell apoptosis **(C, D)**, mitochondrial ROS level **(E, G)**, MMP **(F, H)**, ATP level **(I)** and lncRNA Snhg1 expression **(J)** were detected respectively. (**A-C, G-J**) One-way ANOVA followed by Dunnett’s multiple comparisons test. **P < 0.01, ***P < 0.001 vs. control; ^###^*P* < 0.001 vs. H/R

To clarify the action of lncRNA Snhg1 on MIRI, HL-1 cells were selected to construct the H/R model. The lncRNA Snhg1 level in the H/R model was first measured. It was found that lncRNA Snhg1 was down-regulated after H/R injury and gradually decreased with prolonged re-oxygenation time (Fig. 1A). Therefore, the effects of lncRNA Snhg1 overexpression on H/R-induced injury were next investigated after lncRNA Snhg1 overexpressing lentiviral transduced into HL-1 cells. Results showed that H/R injury decreased the HL-1 cell viability, which was partially restored by enforced lncRNA Snhg1 (Fig. 1B). Besides, H/R injury accelerated HL-1 cell apoptosis but was restrained by overexpressed lncRNA Snhg1 (Fig. 1C and 1D). In addition, mitochondrial ROS level was increased in H/R-stimulated HL-1 cells, while overexpressed lncRNA Snhg1 suppressed this phenomenon (Fig. 1E and 1G). Furthermore, H/R injury decreased the MMP and ATP level of HL-1 cells, which was increased after overexpression of lncRNA Snhg1 (Fig. 1F, 1H and 1I). Moreover, the down-regulated lncRNA Snhg1 caused by H/R challenge was restored by lncRNA Snhg1 overexpressing lentiviral (Fig. 1J). Therefore, overexpressed lncRNA Snhg1 suppressed H/R-stimulated cell apoptosis, mitochondrial ROS production, and mitochondrial polarization in HL-1 cells.

### LncRNA Snhg1 targets miR-361-5p

**Fig. 2:**
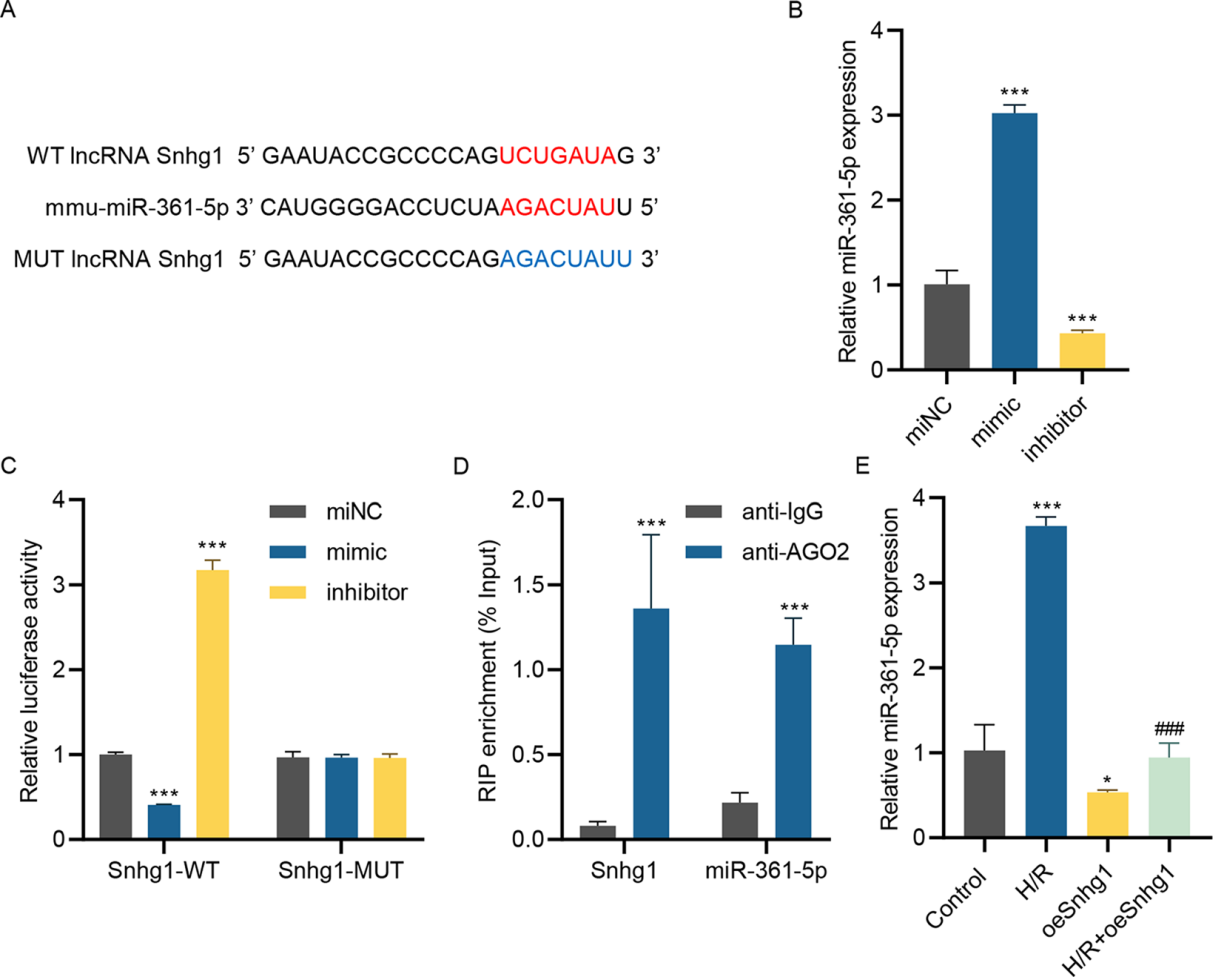
LncRNA Snhg1 targets miR-361-5p. **(A)** The putative target sites between lncRNA Snhg1 and miR-361-5p were presented. **(B)** The miR-361-5p level was measured utilizing RT-qPCR in HL-1 cells introduced into miR-361-5p mimic or inhibitor. **(C)** The association between lncRNA Snhg1 and miR-361-5p was verified using dual-luciferase assay after cells transfected with WT-lncRNA Snhg1 vector or MUT-lncRNA Snhg1 vector and miR-361-5p mimic or inhibitor. **(D)** RIP-PCR detected the binding of lncRNA Snhg1 or miR-361-5p to AGO2. (**E**) The miR-361-5p expression was measured utilizing RT-qPCR in HL-1 cells after pre-transfected with lncRNA Snhg1 overexpressing lentivirus for 24 h and received H/R challenge for 24 h. (**B, C, E**) One-way ANOVA followed by Dunnett’s multiple comparisons test. (**D**) Unpaired *t*-test. *P < 0.05, ***P < 0.001 vs. miNC, IgG, or control; ^###^*P* < 0.001 vs. H/R

LncRNAs usually modulate disease progression by acting as miRNA sponges. Therefore, the target miRNA of lncRNA Snhg1 was screened to investigate the mechanism of lncRNA Snhg1 on MIRI progression. Bioinformatics analysis using Starbase revealed that lncRNA Snhg1 might bind to miR-361-5p, and the presumptive target sites were listed in Fig. 2A. miR-361-5p mimic and inhibitor were introduced into HL-1 cells, which was verified utilizing RT-qPCR (Fig. 2B). After that, the dual-luciferase assay was selected to prove the association of lncRNA Snhg1 and miR-361-5p. Interestingly, miR-361-5p mimic significantly inhibited wild lncRNA Snhg1 activity but had no influence on the mutant lncRNA Snhg1 activity (Fig. 2C). Conversely, miR-361-5p inhibitor greatly enhanced wild lncRNA Snhg1 activity, while it had no action on the mutant lncRNA Snhg1 activity (Fig. 2C). Meanwhile, the RIP experiment presented the significant enrichment of lncRNA Snhg1 and miR-361-5p using the anti-AGO2 antibody compared to the anti-IgG antibody (Fig. 2D). Besides, H/R injury promoted the miR-361-5p level, while overexpressed lncRNA Snhg1 repressed the miR-361-5p level (Fig. 2E). Thus, lncRNA Snhg1 targeted miR-361-5p.

### LncRNA Snhg1 silencing promotes H/R-stimulated cell apoptosis, mitochondrial ROS production, and mitochondrial polarization via targeting miR-361-5p

**Fig. 3:**
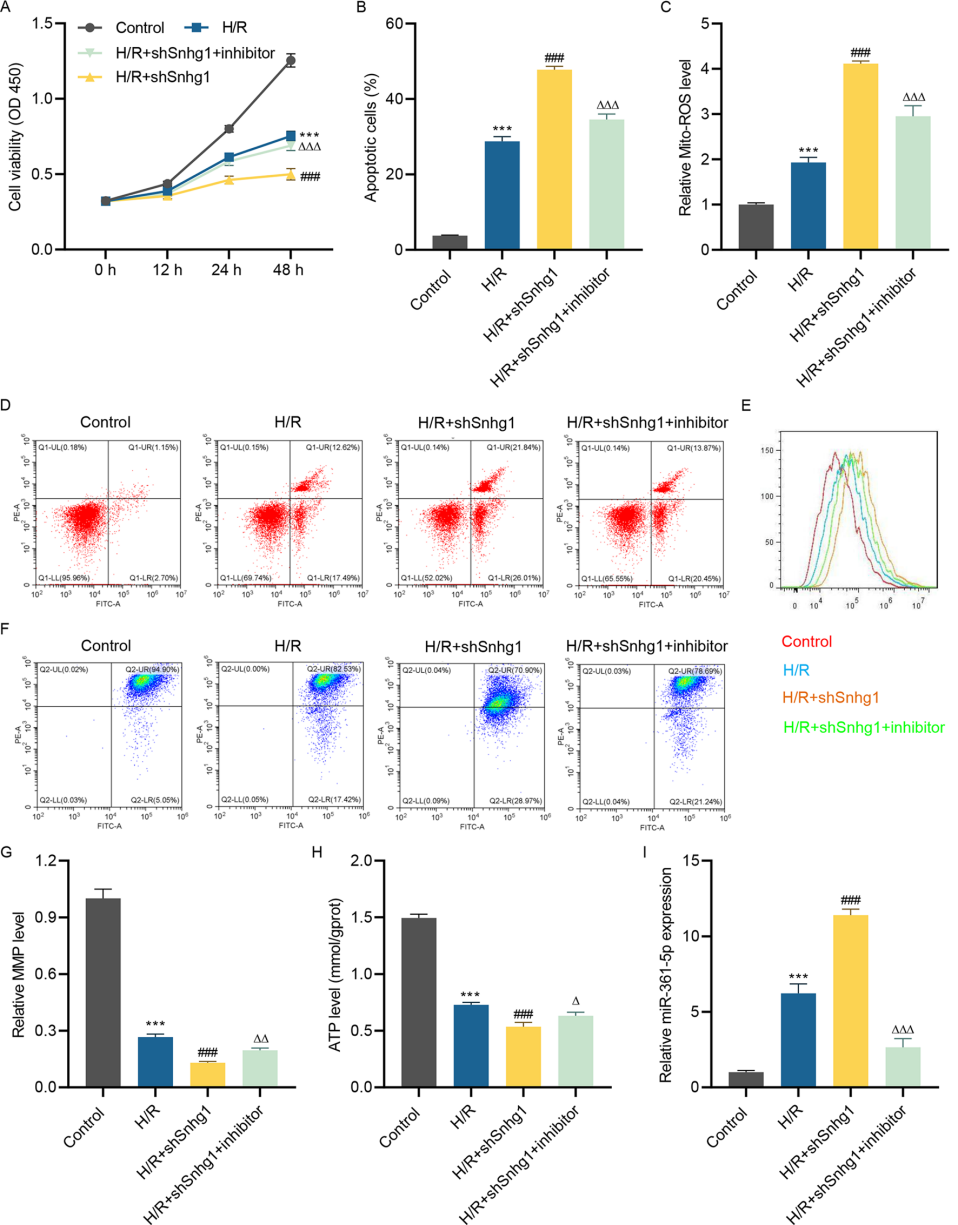
LncRNA Snhg1 silencing promotes H/R-stimulated cell apoptosis, mitochondrial ROS level and polarization by targeting miR-361-5p. After HL-1 cells pre-transfected with shSnhg1 lentivirus with or without miR-361-5p inhibitor for 24 h, followed by receiving H/R challenge for 24 h, cell viability **(A)**, cell apoptosis **(B, D)**, mitochondrial ROS level **(C, E)**, MMP **(F, G)**, ATP level **(H)** and lncRNA Snhg1 expression **(I)** were measured respectively. (**A-C, G-I**) One-way ANOVA followed by Dunnett’s multiple comparisons test. ***P < 0.001 vs. control; ^###^*P* < 0.001 vs. H/R; ^Δ^*P* < 0.05, ^ΔΔ^*P* < 0.01, ^ΔΔΔ^*P* < 0.001 vs. H/R + shSnhg1

To elucidate the precise mechanism of lncRNA Snhg1 on MIRI progression, shSnhg1 lentivirus, and miR-361-5p inhibitor were introduced into H/R-induced HL-1 cells. Results showed that H/R treatment suppressed cell viability of HL-1 cells, and silencing of lncRNA Snhg1 exacerbated H/R-induced injury on cell viability, while this phenomenon was reversed after miR-361-5p inhibitor treatment (Fig. 3A). Besides, the promotion effect of silenced lncRNA Snhg1 on H/R-stimulated cell apoptosis was restrained by miR-361-5p inhibitor (Fig. 3B and 3D). Interestingly, H/R-induced mitochondrial ROS production was facilitated by silenced lncRNA Snhg1, but this action was repressed by miR-361-5p inhibitor (Fig. 3C and 3E). H/R injury decreased the MMP and ATP levels of HL-1 cells, and silenced lncRNA Snhg1 aggravated this effect, which was abolished by miR-361-5p inhibitor (Fig. 3F and 3H). Furthermore, H/R treatment induced the miR-361-5p level, and silencing of lncRNA Snhg1 further increased the miR-361-5p expression, which was abrogated by miR-361-5p inhibitor (Fig. 3I). Hence, silencing of lncRNA Snhg1 promoted H/R-stimulated cell apoptosis, mitochondrial ROS level and polarization in HL-1 cells by targeting miR-361-5p.

### Mir-361-5p targets OPA1

**Fig. 4:**
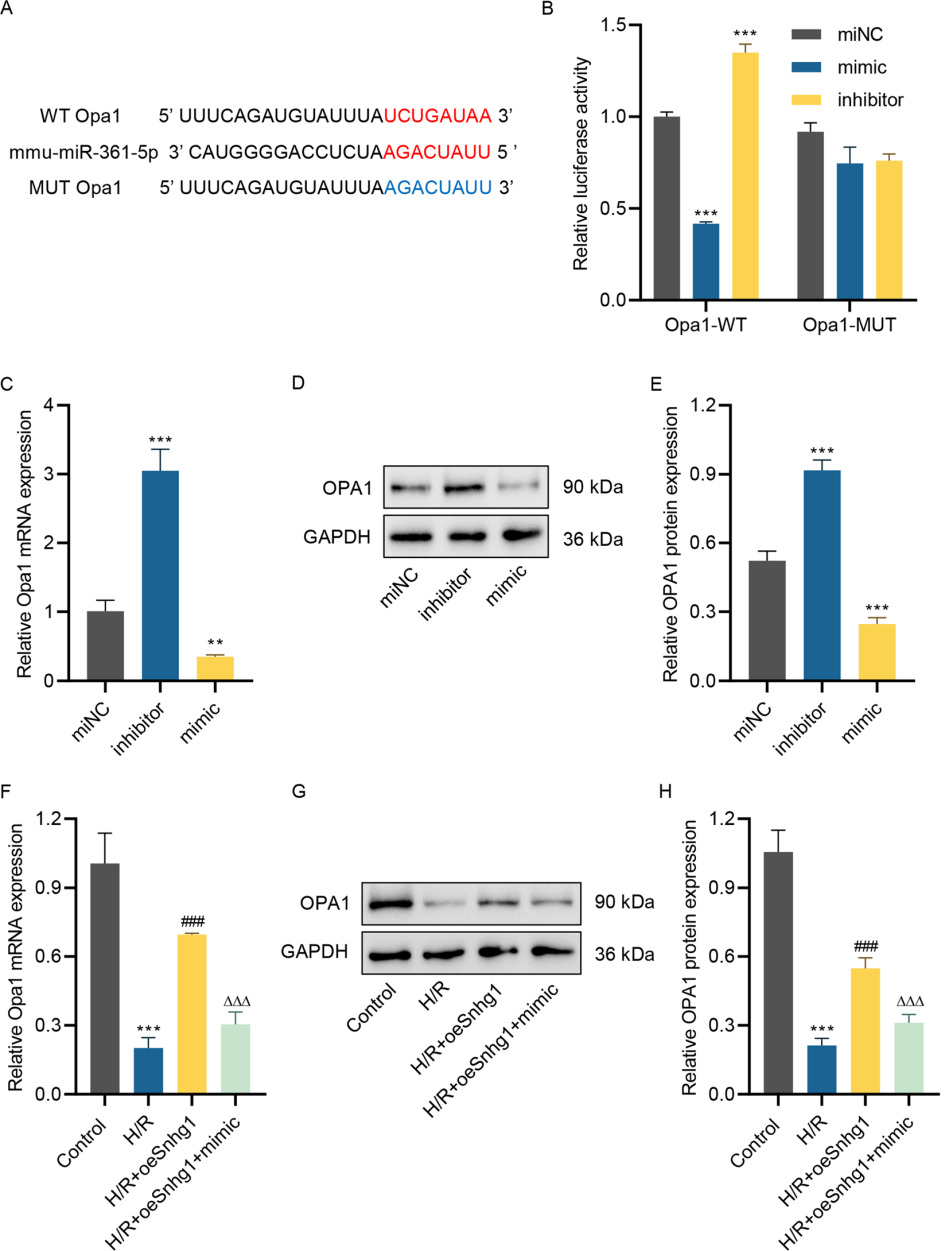
miR-361-5p can target Opa1. **(A)** The putative target sites between miR-361-5p and Opa1 were exhibited. **(B)** The relation of miR-361-5p and Opa1 was validated utilizing dual-luciferase assay after cells introduced into WT-3’-UTR of Opa1 vector or MUT-3’-UTR of Opa1 vector and miR-361-5p mimic or inhibitor. **(C)** The Opa1 mRNA level was measured using RT-qPCR after HL-1 cells introduced into miR-361-5p mimic or inhibitor. **(D, E)** The Opa1 protein level was evaluated utilizing western blot after HL-1 cells introduced into miR-361-5p mimic or inhibitor. **(F)** The Opa1 mRNA level was measured using RT-qPCR after HL-1 cells pre-transfected with miR-361-5p mimic with or without lncRNA Snhg1 overexpression lentivirus for 24 h and then received H/R challenge for 24 h. **(G, H)** The Opa1 protein level in HL-1 cells was assessed utilizing western blot after pre-transfected with miR-361-5p mimic with or without lncRNA Snhg1 overexpression lentivirus for 24 h and then received H/R challenge for 24 h. (**B, C, E, F, H**) One-way ANOVA followed by Dunnett’s multiple comparisons test. **P < 0.01, ***P < 0.001 vs. miNC or control; ^###^*P* < 0.001 vs. H/R; ^ΔΔΔ^*P* < 0.001 vs. H/R + oeSnhg1

MiRNAs generally exerted effects via targeting mRNAs. Therefore, the target mRNAs of miR-361-5p were searched. Bioinformatics analysis using TargetScan discovered that miR-361-5p might target Opa1, and the presumptive target sites were listed in Fig. 4A. Dual-luciferase assay found that miR-361-5p mimic significantly restrained the activity of wild Opa1 3’-UTR but had no impact on the activity of mutant Opa1 3’-UTR (Fig. 4B). Instead, miR-361-5p inhibitor amplified the activity of wild Opa1 3’-UTR but did not influence the activity of mutant Opa1 3’-UTR (Fig. 4B). Besides, miR-361-5p mimic restrained the OPA1 levels, but miR-361-5p inhibitor increased the OPA1 levels (Fig. 4C and 4E). In addition, H/R treatment decreased the level of OPA1, while overexpressed lncRNA Snhg1 increased the OPA1 level (Fig. 4F and 4H). However, the promotion effect of overexpressed lncRNA Snhg1 on OPA1 level in H/R-stimulated HL-1 cells was abrogated by miR-361-5p mimic (Fig. 4F and 4H). Taken together, miR-361-5p could target OPA1.

### miR-361-5p inhibitor inhibits H/R-stimulated cell apoptosis, mitochondrial ROS production, and mitochondrial polarization in HL-1 cells by targeting OPA1

**Fig. 5:**
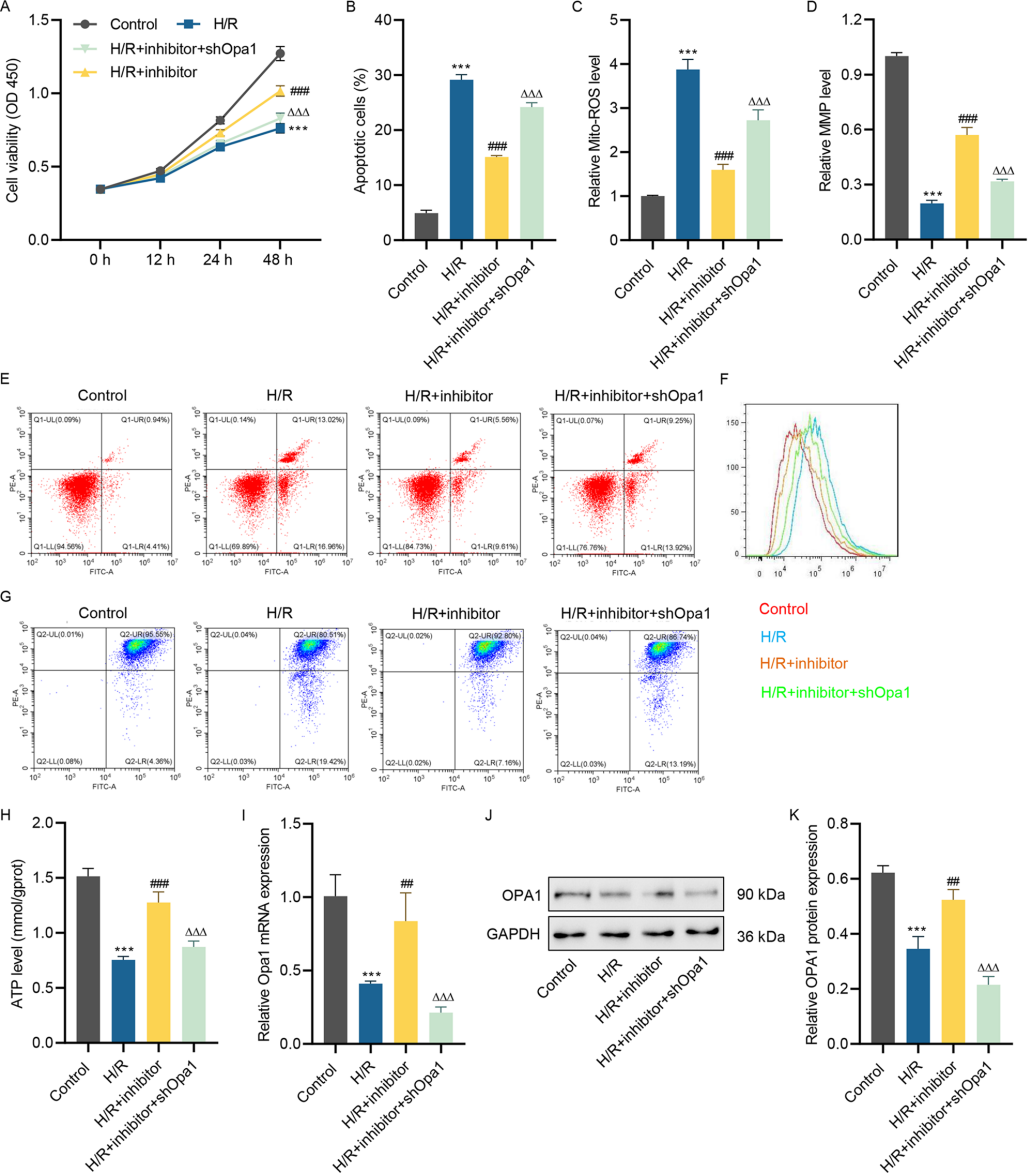
miR-361-5p inhibitor inhibits H/R-stimulated cell apoptosis, mitochondrial ROS level and polarization in HL-1 cells by targeting Opa1. After HL-1 cells pre-transfected with shOpa1 lentivirus with or without miR-361-5p inhibitor for 24 h and received H/R challenge for 24 h, cell viability **(A)**, cell apoptosis **(B, E)**, mitochondrial ROS level **(C, F)**, MMP **(D, G)**, ATP level **(H)** and Opa1 mRNA **(I)** and protein levels **(J, K)** were measured respectively. (**A-D, H, I, K**) One-way ANOVA followed by Dunnett’s multiple comparisons test. ***P < 0.001 vs. control; ^##^*P* < 0.01, ^###^*P* < 0.001 vs. H/R; ^ΔΔΔ^*P* < 0.001 vs. H/R + inhibitor

To elucidate whether miR-361-5p regulates MIRI development, miR-361-5p inhibitor, and shOpa1 lentivirus was introduced into H/R-induced HL-1 cells. It was presented that H/R injury suppressed HL-1 cell viability, and miR-361-5p inhibitor improved H/R-induced damage on cell viability, which was abolished by silenced Opa1 (Fig. 5A). Besides, the suppression action of miR-361-5p inhibitor on H/R-induced cell apoptosis was abolished by silenced Opa1 (Fig. 5B and E). In addition, H/R-induced mitochondrial ROS level was inhibited by miR-361-5p inhibitor, but silenced Opa1 reversed this effect (Fig. 5C and F). Furthermore, H/R injury decreased the MMP and ATP levels of HL-1 cells, and miR-361-5p inhibitor increased these levels, which was abrogated by silenced Opa1 (Fig. 5D, G and H). Moreover, H/R treatment inhibited the OPA1 levels, and miR-361-5p inhibitor increased the OPA1 level, which was abrogated by silenced Opa1 (Fig. 5I and K). Collectively, miR-361-5p inhibitor inhibits H/R-stimulated cell apoptosis, mitochondrial ROS level and polarization in HL-1 cells by targeting OPA1.

### WTAP induces lncRNA Snhg1 m^6^A modification

**Fig. 6:**
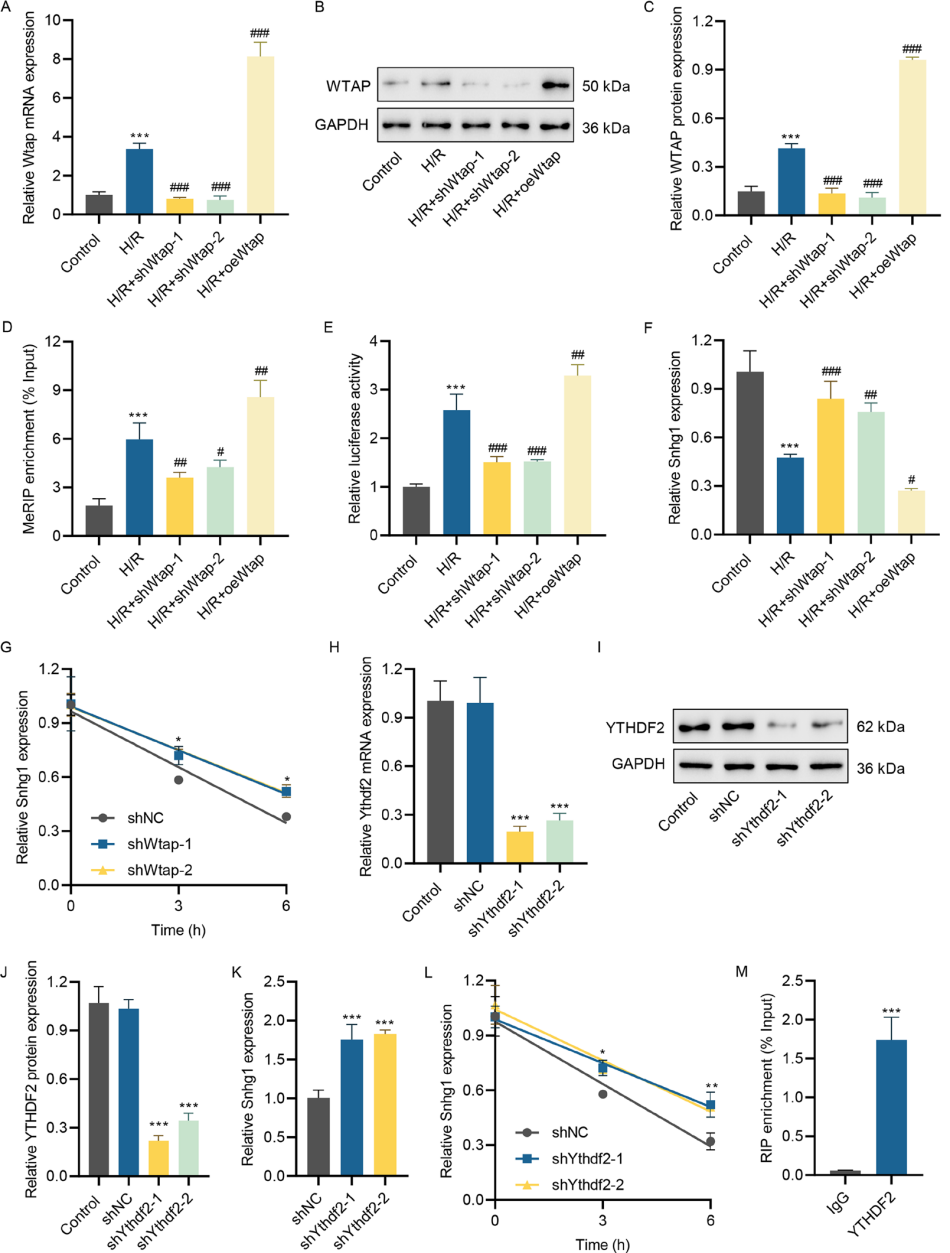
WTAP induces lncRNA Snhg1 m^6^A modification**(A)** The WTAP mRNA expression was detected utilizing RT-qPCR after HL-1 cells introduced into shWtap lentivirus or WTAP overexpression lentivirus and received H/R challenge for 24 h. **(B, C)** The Wtap level was assessed utilizing western blot after HL-1 cells transfected with shWtap lentivirus or Wtap overexpression lentivirus and treated with H/R for 24 h. **(D)** LncRNA Snhg1 m^6^A level was detected using MeRIP-PCR after HL-1 cells transfected with shWtap lentivirus or Wtap overexpression lentivirus and received H/R challenge for 24 h. **(E)** The lncRNA Snhg1 m^6^A activity was tested utilizing luciferase assay after HL-1 cells transfected with shWtap lentivirus or Wtap overexpression lentivirus and treated with H/R for 24 h. **(F)** The lncRNA Snhg1 level in HL-1 cells introduced into shWtap lentivirus or Wtap overexpression lentivirus and received H/R challenge for 24 h was evaluated utilizing RT-qPCR. **(G)** LncRNA Snhg1 level in HL-1 cells introduced into shWtap lentivirus and incubated with actinomycin D for 3 h and 6 h was evaluated utilizing RT-qPCR. **(H)** The YTHDF2 mRNA level was measured in HL-1 cells after introduced into shYthdf2 lentivirus for 48 h using RT-qPCR. **(I, J)** The YTHDF2 protein level was assessed in HL-1 cells after introduced into shYthdf2 lentivirus for 48 h using western blot. **(K)** The level of lncRNA Snhg1 in HL-1 cells after introduced into shYthdf2 lentivirus for 48 h was assessed utilizing RT-qPCR. **(L)** The lncRNA Snhg1 level in HL-1 cells after transfected with shYthdf2 lentivirus and incubated with actinomycin D for 3 h and 6 h was evaluated utilizing RT-qPCR. **(M)** The combination of YTHDF2 and lncRNA Snhg1 m^6^A site was determined using RIP-PCR. (**A, C-H, J-L**) One-way ANOVA followed by Dunnett’s multiple comparisons test. (M) Unpaired *t*-test. *P < 0.05, **P < 0.01, ***P < 0.001 vs. control, shNC, or IgG; ^#^*P* < 0.05, ^##^*P* < 0.01, ^###^*P* < 0.001 vs. H/R

To illustrate whether lncRNA Snhg1 was modified by m^6^A methylation, the shWtap lentivirus and Wtap overexpression lentivirus were introduced into H/R-induced HL-1 cells, which restrained and enhanced the Wtap level, respectively (Fig. 6A and 6C). MeRIP-PCR assay proved the elevated m^6^A level of lncRNA Snhg1 in H/R-stimulated HL-1 cells, while silenced Wtap drastically decreased the m^6^A level of lncRNA Snhg1 (Fig. 6D). Conversely, overexpressed Wtap increased the m^6^A level of lncRNA Snhg1 (Fig. 6D). Luciferase assay showed that lncRNA Snhg1 m^6^A activity was enhanced in H/R-induced HL-1 cells, while silenced Wtap drastically decreased the lncRNA Snhg1 m^6^A activity (Fig. 6E). Overexpressed WTAP increased the lncRNA Snhg1 m^6^A activity (Fig. 6E). After that, whether m^6^A modification on lncRNA Snhg1 affected its expression was determined. It was observed that silenced Wtap enhanced the lncRNA Snhg1 expression, but enforced Wtap suppressed the lncRNA Snhg1 expression (Fig. 6F). Besides, the RNA stability of lncRNA Snhg1 was measured after silenced Wtap and treated with actinomycin D. Results showed that silenced Wtap increased the stability of lncRNA Snhg1 (Fig. 6G). Additionally, the effect of m^6^A “reader” YTHDF2 on lncRNA Snhg1 expression was studied after transfection of shYthdf2. The shYthdf2 considerably silenced the Ythdf2 expression (Fig. 6H and 6J). The lncRNA Snhg1 was elevated after silencing Ythdf2 (Fig. 6K). Silenced Ythdf2 increased the stability of lncRNA Snhg1 (Fig. 6L). Furthermore, the RIP-PCR assay found that lncRNA Snhg1 was obviously enriched using the anti-YTHDF2 antibody (Fig. 6M). Overall, these results suggested that WTAP induced lncRNA Snhg1 m^6^A modification.

### LncRNA Snhg1 overexpression inhibits I/R-induced myocardial tissue damage and apoptosis

**Fig. 7:**
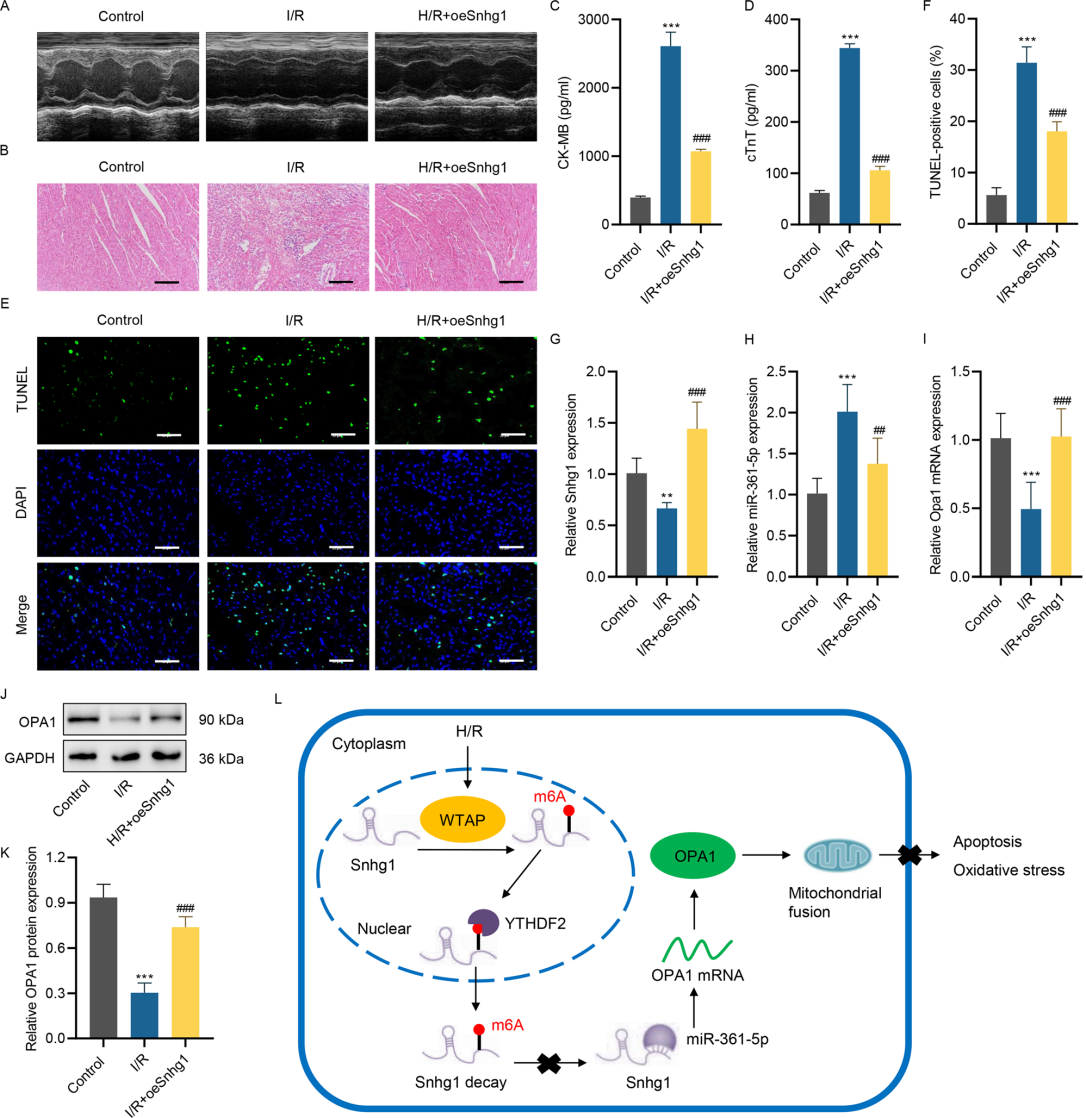
LncRNA Snhg1 overexpression inhibits I/R-stimulated myocardial tissue damage and apoptosis. **(A)** Representative echocardiographic images. **(B)** HE staining was conducted in myocardial tissue of I/R mice pretreated with lncRNA Snhg1 overexpression lentivirus. Scale bar, 200 μm. (**C, D**) The serum levels of CK-MB and cTnT were evaluated by ELISA in I/R mice pretreated with lncRNA Snhg1 overexpression lentivirus **(E, F)** Myocardial tissue apoptosis was evaluated using TUNEL assay in I/R mice pretreated with lncRNA Snhg1 overexpression lentivirus. Scale bar, 100 μm. The lncRNA Snhg1 **(G)**, miR-361-5p **(H)**, and Opa1 mRNA levels **(I)** in I/R mice pretreated with lncRNA Snhg1 overexpression lentivirus were tested utilizing RT-qPCR. **(J, K)** The Opa1 level in I/R mice pretreated with lncRNA Snhg1 overexpression lentivirus was detected utilizing western blot. (**L**) The mechanism diagram revealing the regulation of WTAP-mediated m6A modification of lncRNA Snhg1 improved on myocardial ischemia-reperfusion injury via miR-361-5p/Opa1-dependent mitochondrial fusion. (**C, D, F-I, K**) One-way ANOVA followed by Dunnett’s multiple comparisons test. **P < 0.01, ***P < 0.001 vs. control; ^##^*P* < 0.01, ^###^*P* < 0.001 vs. I/R

To interpret the action of lncRNA Snhg1 on MIRI progression in vivo, the myocardial I/R mouse model was established with overexpressed lncRNA Snhg1. Echocardiography was performed to measure cardiac function parameters (Fig. 7A). Cardiac functions results showed that I/R treatment significantly reduced cardiac ejection fraction, fractional shortening, left ventricular end systolic pressure and heart rate, and promoted left ventricular end diastolic pressure (*P* < 0.001), these phenomena were reversed by overexpressed lncRNA Snhg1 (*P* < 0.001, Table 2). HE staining results found that the myocardial tissue exhibited apparent damage, including disordered structure, inflammatory infiltration, and interstitial edema after I/R treatment, which was alleviated by overexpressed lncRNA Snhg1 (Fig. 7B). The serum levels of CK-MB and cTnT were also increased in I/R mice, which were alleviated by overexpressed lncRNA Snhg1 (Fig. 7C and 7D). Besides, I/R treatment induced myocardial cell apoptosis, but overexpressed lncRNA Snhg1 suppressed this effect (Fig. 7E and 7F). In addition, the lncRNA Snhg1 expression was decreased in myocardial tissue after I/R injury, which was elevated after transfection of lncRNA Snhg1 overexpression lentivirus (Fig. 7G). Furthermore, miR-361-5p was enhanced in the myocardial tissue of I/R mice, and overexpressed lncRNA Snhg1 restrained the miR-361-5p level (Fig. 7H). Moreover, OPA1 were repressed in myocardial tissue after I/R treatment, which was restored by overexpressed lncRNA Snhg1 (Fig. 7I and 7K). To sum up, enforced lncRNA Snhg1 restrained I/R-stimulated myocardial tissue damage and apoptosis and regulated the miR-361-5p and OPA1 levels.

**Table 2:** Cardiac functions for experiment animals

Group	Echocardiographic data (%)		Left ventricular end pressure (mmHg)		Heart rate (beat/min)
	EF	FS	Systolic	Diastolic	
Control	86.12±1.40	48.27±1.76	103.3±1.33	7.83±0.75	367.67±17.43
I/R	59.28±5.13^a^	25.99±3.11^a^	75.07±1.45^a^	12.43±0.16^a^	273.00±10.20^a^
I/R + oeSnhg1	72.23±3.45^b^	34.85±2.66^b^	83.52±2.25^b^	9.54±0.80^b^	313.33±13.25^b^

EF, ejection fraction; FS, fractional shortening. ^a^*P*<0.001 compared with control group; ^b^*P*<0.001 compared with I/R

## Discussion

Myocardial ischemia-reperfusion injury (MIRI) is an injury caused by the recovery of coronary artery blood flow after ischemic heart disease and increases myocardial infarction [3–6]. LncRNA Snhg1 regulates disease progression by modulating the cell cycle, apoptosis, autophagy, oxygen species, and mitochondrial function [33–35]. N6-methyladenosine (m^6^A) is the frequent RNA modification manner, and lncRNAs can be modified by m^6^A methylation [22, 24]. In this research, we elucidated the action and mechanism of lncRNA Snhg1 on MIRI progression and clarified whether the lncRNA Snhg1 was modified by m^6^A methylation. The findings revealed that WTAP-mediated m^6^A modified lncRNA Snhg1 modulated MIRI progression manifested in regulating cell apoptosis, mitochondrial ROS production, and MMP via miR-361-5p/OPA1 axis (Fig. 7L). These results demonstrated the existence of the WTAP/SNHG1/miR-361-5p/OPA1 regulatory axis and provided a novel therapeutic target for the treatment of ischemic cardiomyocyte injury.

LncRNA Snhg1 belongs to the lncRNAs family, and it usually presents abnormal expression in various disease tissues and cells [36–38]. To elaborate the potential action of lncRNA Snhg1 on MIRI, the lncRNA Snhg1 level was explored in H/R-stimulated HL-1 cells and found that lncRNA Snhg1 was decreased after H/R challenge, which was in line with the reported study [18]. The abnormal level of lncRNA Snhg1 implied possible involvement in MIRI progression. As expected, overexpression of lncRNA Snhg1 restored HL-1 cell viability inhibited by H/R challenge. Actually, the regulatory action of lncRNA Snhg1 on cell viability has also been demonstrated in multiple disorders, such as cancers, Parkinson’s disease, and hypoxic injury [38–40]. Besides, apoptosis is a significant feature of MIRI and is considered an essential pathogenic factor in MIRI [3, 41]. Apoptosis causes cardiomyocyte loss and myocardium remolding and aggravates inflammatory response [3]. Therefore, inhibiting myocardial cell apoptosis is critical for limiting MIRI progression [41]. This research demonstrated that enforced lncRNA Snhg1 restrained myocardial cell apoptosis in H/R-stimulated HL-1 cells and I/R mice model. Similarly, overexpressed lncRNA Snhg1 was also proved to repress cell apoptosis behavior in H/R-stimulated AC16 cells [18]. Lv et al. revealed that enforced lncRNA Snhg1 inhibited oxygen-glucose deprivation-stimulated cell apoptosis [42]. Furthermore, it is accepted that mitochondria are key targets and the source of tissue damage, and the structural and functional changes of mitochondria are critical in the pathogenesis of MIRI [10]. MIRI could cause early mitochondrial-driven injury with excessive ROS production and calcium regulation disorder, causing abnormal permeation, loss of MMP, and swelling and damage to mitochondria of mitochondrial permeability transition pore [43, 44]. In the current study, increased mitochondrial ROS production and decreased MMP and ATP levels were exhibited after H/R challenge, which was in line with the reported research [10]. Interestingly, these changes in the structural and functional changes of mitochondria caused by H/R challenge were reversed by overexpressed lncRNA Snhg1. In other words, enforced lncRNA Snhg1 suppressed mitochondrial ROS production and mitochondrial polarization in H/R-induced HL-1 cells. To sum up, these findings indicated the effectiveness of lncRNA Snhg1 in improving cell apoptosis, mitochondrial ROS production, and mitochondrial polarization in MIRI progression.

LncRNAs generally regulate disease progression via the ceRNA mechanism [14]. To elucidate the precise mechanism of lncRNA Snhg1 on the regulation of cell apoptosis, mitochondrial ROS production, and mitochondrial polarization in MIRI progression, the potential target miRNA of lncRNA Snhg1 was searched, and the findings discovered that lncRNA Snhg1 could target miR-361-5p. So far, this study first reports that lncRNA Snhg1 targeted miR-361-5p. The actions of miR-361-5p on modulating cardiomyocyte apoptosis and mitochondrial function were proved in previous studies [45, 46]. Li *et al*. proved that inhibiting miR-361-5p expression limited cardiomyocyte apoptosis in the mouse model of sepsis-stimulated myocardial injury [45]. Wang *et al*. clarified that decreased miR-361-5p provoked the reduction of mitochondrial fission in mice with cardiac ischemia [46]. The miRNAs usually exerted functions via inhibiting the target gene expression through binding to 3’-UTR [47]. Therefore, the target gene of miR-361-5p was explored, and the findings demonstrated that miR-361-5p could target OPA1. OPA1, a GTPase at the mitochondrial inner membrane, exerts a critical function on mitochondrial fusion [12]. As expected, the miR-361-5p inhibitor inhibited H/R-stimulated cell apoptosis, mitochondrial ROS level and polarization in HL-1 cells by targeting OPA1. Consistent with our findings, the previous study reported that OPA1 was reduced in cardiomyocytes with simulated I/R challenge, and depletion of OPA1 sensitized the cells to mitochondrial fragmentation, apoptosis, and I/R injury [48]. Wang *et al*. also found that OPA1 attenuated ROS production and mitochondrial dysfunction in H_2_O_2_-challenged H9C2 cells [49]. Besides, we also found overexpressed lncRNA Snhg1 restrained the miR-361-5p expression and elevated OPA1 level in the I/R mice model in vivo. Overall, this evidence suggested that lncRNA Snhg1 regulated MIRI progression via the miR-361-5p/OPA1 axis.

The m^6^A methylation is the frequent RNA modification manner in eukaryotic cells [20, 21]. Recent studies discovered that lncRNAs could be modified by m^6^A methylation [22, 24]. Jiang et al. reported that METTL3 induced the lncRNA Snhg1 m^6^A modification and improved the stability of the lncRNA Snhg1 in non-small cell lung cancer [50]. Therefore, whether lncRNA Snhg1 was modified by m^6^A methylation in HL-1 cells after H/R challenge was studied in this study and it was proved that WTAP induced lncRNA Snhg1 m^6^A modification and YTHDF2 was a specific m^6^A reader of lncRNA Snhg1 in HL-1 cells with H/R challenge. These findings suggested that WTAP-mediated m^6^A is associated with the expression of lncRNA Snhg1, probably through regulating lncRNA Snhg1 stability, which provided new answers to the question of why lncRNA Snhg1 exhibited abnormal expression after H/R injury.

HL-1 cardiomyocytes are currently the only cells available that continuously divide, spontaneously contract, and maintain a differentiated adult cardiac phenotype through indefinite passages in culture [51]. HL-1 cells have also been used to address pathological conditions such as apoptosis, hypoxia, and I/R [52]. However, the expression patterns of cardiomyocyte markers and whole transcriptomic profile indicate low-to-moderate similarity of HL-1 cells to primary cells/cardiac tissues [53]. Therefore, we will validate the role of lncRNA SNHG1/miR-361-5p/OPA1 axis in MIRI using primary myocardial cells in future experiments. In addition to lncRNA SNHG1/miR-361-5p/OPA1, there are many lncRNA SNHG1/miRNA/mRNA-mediated regulatory pathways of myocardial injury, such as lncRNA SNHG1/miR-137-3p/KLF4 [16], lncRNA SNHG1/miR‑16‑5p/GATA4 [17] and SNHG1/miR‑450b‑5p/IGF1 [18]. Thus the effect of these lncRNA SNHG1-mediated and other reported mechanisms still need further research and evidence.

## Conclusions

WTAP-mediated m^6^A modification of lncRNA Snhg1 regulated MIRI progression through modulating myocardial apoptosis, mitochondrial ROS production, and mitochondrial polarization via the miR-361-5p/Opa1 axis, providing the evidence for lncRNA as the prospective target for alleviating MIRI progression.

## Data Availability

The datasets used and/or analysed during this study were accessed via the corresponding authors on reasonable request.

## Abbreviations

MIRI: Myocardial ischemia-reperfusion injury
m6A: N6-methyladenosine
H/R: hypoxia/reoxygenation
ROS: reactive oxygen species
MMP: mitochondrial membrane potential
IHD: Ischemic heart disease
lncRNAs: Long non-coding RNAs
ceRNAs: competing endogenous RNAs
miRNAs: microRNAs
RT-qPCR: Quantitative real-time PCR
RIP: RNA immunoprecipitation
HE: hematoxylin and eosin
TUNEL: terminal-deoxynucleoitidyl transferase mediated nick end labeling
